# Participation in prevention measures during the corona pandemic in Germany in 2020/21: Susanne Jordan

**DOI:** 10.1093/eurpub/ckac130.136

**Published:** 2022-10-25

**Authors:** S Jordan, R Kuhnert, N Schmid-Küpke, A Starker

**Affiliations:** 1 Department of Epidemiology and Health Monitoring, Robert Koch Institute, Berlin, Germany; 2 Department of Infectious Disease Epidemiology, Robert Koch Institute, Berlin, Germany

## Abstract

**Background:**

The availability and use of preventive measures such as diet courses, sports groups, and counselling services were hindered by containment measures set by the German government and local authorities to reduce the spread of the COVID-19 pandemic. Regulations on contact restriction, closure of sport and leisure facilities made it difficult to use prevention programs in 2020 and 2021. So far, no information is available to what extent the participation of the population in prevention programs has changed as a result of the pandemic and whether there are group differences regarding socio-demographic characteristics.

**Methods:**

We used standardized telephone interviews of the adult German-speaking population to ask for changes in the participation in prevention measures in the last 12 months as a result of the corona pandemic. The data were collected between 17.3.2021 and 18.08.2021 in four cross-sectional surveys and is representative of the population aged 18 years and older in Germany. Analyses of the weighted and pooled data were conducted for n = 3,998 individuals by gender, age and education.

**Results:**

Almost one third of the respondents reported a lower use of programs (28.3%). An unchanged use of the programs was stated by 6.5%, a higher use by 2.1%. 63.2% said they did not use such programs. More women (33.6%) than men (22.7%) reported reduced pandemic-related use (p < 0.0001). For women, we observed a difference between age and education groups, but not for men: Lower participation was reported by a higher proportion of older than younger women (p < 0.0001). More women with high education reported an increased use than ones with low education (p = 0.003) (preliminary results).

**Conclusions:**

The observed differences indicate to different barriers to use prevention measures during the pandemic, especially for women. To promote equal opportunities, resilient structures of prevention and health promotion should be built for future crises in advance.

**Key messages:**

- The use of prevention measures was seriously reduced by the corona pandemic.
- To be prepared for further crises, concrete barriers to use prevention programs under crisis conditions should be identified and addressed through public health actions.

